# Targeted LC-MS/MS method for quantifying respiratory pharmaceuticals in wastewater

**DOI:** 10.1039/d5ew00894h

**Published:** 2025-11-28

**Authors:** Regina L. Gasparetto, Scott Bickel, Xinmin Yin, Ted Smith, Aruni Bhatnagar, Rochelle H. Holm, Xiang Zhang

**Affiliations:** a Department of Chemistry, University of Louisville 2210 South Brook Street Louisville Kentucky 40208 USA xiang.zhang@louisville.edu +1 502 852 8878; b Center for Regulatory and Environmental Analytical Metabolomics, University of Louisville Louisville Kentucky 40292 USA; c Department of Pediatrics, University of Louisville Louisville Kentucky 40202 USA; d Christina Lee Brown Environment Institute, School of Medicine, University of Louisville Louisville Kentucky 40202 USA rochelle.holm@louisville.edu +1 502 852 5873

## Abstract

*Background*: Wastewater-based epidemiology (WBE) enables the population-level surveillance of molecular and chemical targets. Despite the high prevalence of respiratory diseases, there is a lack of sensitive analytical methods for detecting associated medications in complex wastewater matrices. *Methods*: We developed and validated a liquid chromatography-mass spectrometry (LC-MS)/MS method using multiple reaction monitoring for 10 common respiratory pharmaceuticals. The workflow integrated freeze-drying for preconcentration, online solid-phase extraction for cleanup, and stable isotope-labeled internal standards (SILs) to compensate for matrix effects. *Results*: Detection and quantification limits ranged from 0.7 to 19 ng L^−1^ and 3 to 125 ng L^−1^, respectively, with recoveries of 82–194% and precision within 0.14–7.2% relative standard deviation. Matrix effects (64–228%) were effectively corrected using SILs. Application to 12 neighborhood-level wastewater samples detected 9 of the 10 target compounds, with 6 (albuterol, amoxicillin, azithromycin, cetirizine, diphenhydramine, and fexofenadine), detected above their quantification limits. Fexofenadine was the most abundant, reaching 3309 ng L^−1^. *Conclusion*: This robust, low-volume, high-throughput LC-MS/MS method enables the reliable detection of respiratory pharmaceuticals in wastewater, supporting WBE applications for pharmaceutical use surveillance.

## Abstract 2

*Background*: We developed and validated a liquid chromatography-mass spectrometry (LC-MS)/MS method using multiple reaction monitoring for 10 common respiratory pharmaceuticals.

## Graphical abstract

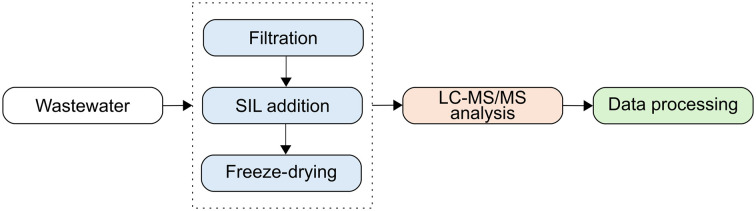

**Water impact**

Respiratory diseases are highly prevalent, yet sensitive analytical methods are needed to detect associated medications in complex wastewater matrices. We developed and validated a high-throughput LC-MS/MS method optimized for detecting 10 pharmaceuticals frequently prescribed for common respiratory conditions: albuterol, amoxicillin, azithromycin, budesonide, cetirizine, diphenhydramine, fexofenadine, fluticasone propionate, prednisolone, and prednisone.

## 1. Introduction

Wastewater-based epidemiology (WBE) has emerged as a reliable approach for community surveillance for both molecular (*e.g.*, pathogens) and chemical (*e.g.*, pharmacologic compounds and their metabolites) targets.^1–3^ Beyond its scientific utility, WBE has gained strong public support, particularly for surveillance of prescription medication usage.^4^ Respiratory diseases, including asthma, chronic obstructive pulmonary disease, allergic rhinitis, sinus infections, and pneumonia, are among the most common conditions requiring pharmacological treatment. In the United States, these diseases impose a substantial burden on healthcare resources.^5^ Their incidence is strongly influenced by environmental exposures^6^ and are often associated with inconsistent prescribing practices, particularly the use of antibiotics for viral respiratory infections. Inappropriate use of antibiotics increases the risk of antimicrobial resistance, which is a major public health concern.^7^ Reliable surveillance of population-level pharmaceutical usage in specific geographic areas could provide valuable insights into prescribing practices, disease trends, and environmental influences across neighborhoods with differing environmental conditions. Moreover, correlating pharmaceutical concentrations with viral loads in wastewater may enhance outbreak surveillance.

Wastewater is an effective medium for community-level surveillance, as many pharmaceutical biomarkers are excreted in urine and feces.^8,9^ However, quantifying these compounds remains challenging due to analyte stability, sample collection, storage conditions, and analytical performance.^9^

To address the aforementioned challenges, most existing methods use offline solid-phase extraction (SPE) for sample cleanup and enrichment, followed by liquid chromatography-mass spectrometry (LC-MS/MS analysis).^10–16^ Although offline SPE is effective, it introduces variability, increases sample loss, requires large sample volumes, and is both time-consuming and labor-intensive. Additionally, co-extracted matrix components may cause ion suppression, compromising quantification accuracy.^17^ Stable isotope-labeled molecules as internal standards (SILs) are used to mitigate matrix effects because they behave similarly to their unlabeled counterparts, thereby improving the quantification reliability.^14,18,19^ Direct injection MS has also been explored as an SPE alternative,^17,20–22^ but it remains inadequate owing to its poor sensitivity for low-abundance molecules in complex wastewater matrices.^23^

Although previous studies have conducted community surveillance for medications, the current method needs to be improved. This study aimed to develop and validate a high-throughput LC-MS/MS method optimized for detecting 10 pharmaceuticals frequently prescribed for common respiratory conditions: albuterol, amoxicillin, azithromycin, budesonide, cetirizine, diphenhydramine, fexofenadine, fluticasone propionate, prednisolone, and prednisone. To our knowledge, this is the first method to incorporate freeze-drying for preconcentration, online SPE for sample cleanup and reduced sample losses, and multiple reaction monitoring (MRM) for robust quantification. Integration of these analytical approaches increases the probability of detecting low-abundance molecules. The method was validated using 10 pharmaceutical standards, and SILs were incorporated to compensate for matrix effects, thereby improving quantification. Finally, the applicability of the method was demonstrated through an analysis of 12 *in situ* wastewater samples.

## 2. Materials and methods

### 2.1 Chemicals and reagents

The ten target pharmaceuticals (Table 1), namely, albuterol, amoxicillin, azithromycin, budesonide, cetirizine, diphenhydramine, fexofenadine, fluticasone propionate, prednisolone, and prednisone, were purchased from Sigma-Aldrich Corp. (St. Louis, MO, USA). SILs albuterol-d4, amoxicillin-13C6, azithromycin-13Cd3, budesonide-d8, cetirizine-d8, diphenhydramine-d6, fexofenadine-d10, and fluticasone propionate-d5 were purchased from Toronto Research Chemicals (Toronto, ON, Canada). Prednisolone-d6 and prednisone-d8 were purchased from CDN Isotopes (Pointe-Claire, QC, Canada). LC-MS-grade acetonitrile and formic acid were purchased from Fisher Scientific (Waltham, MA, USA). Analytical-grade water was prepared using a Millipore Synergy UV System (Burlington, MA, USA). Individual stock solutions of each standard were prepared at 1 mg mL^−1^ and stored at −80 °C. Working solutions (10 μg mL^−1^) were prepared by diluting the stock solutions in methanol.

**Table 1:** Target pharmaceuticals associated with common respiratory medications, typical clinical use, and whether prescription or an over the counter medication

Category	Medication/biomarker	CAS number	Typical clinical use	Prescription or OTC
Antihistamines	Cetirizine	83881-52-1	Daily or as needed for allergies	OTC
	Diphenhydramine	88637-37-0		
	Fexofenadine	153439-40-8		
Common oral antibiotics	Amoxicillin	61336-70-7	5–14 day acute course for bacterial infections	Prescription
	Azithromycin	117772-70-0	5 day acute course for bacterial infections	
Inhaled corticosteroids	Budesonide	51333-22-3	Daily inhaled steroid for asthma or nasal steroid for allergies as needed for asthma symptoms	Prescription/OTC^a^
	Fluticasone propionate	80474-14-2		
Short-acting beta-agonists	Albuterol	18559-94-9		Prescription
Systemic corticosteroids	Prednisolone	50-24-8	∼5–7 day course for acute asthma exacerbations	Prescription
	Prednisone	53-03-2		

a Prescription for asthma inhaler; OTC for intranasal administration for allergic rhinitis.

### 2.2 Wastewater sample collection

Composite wastewater samples were collected during 2022–2023 from four streetline utility holes in Louisville, Kentucky, USA. Each sample represented the influent from a catchment population of 1400–5100 individuals. Twenty-four-hour composite samples were collected using automated samplers, and two 125 mL aliquots were obtained from each site. Flow rate data were unavailable. The samples were transported on ice to the University of Louisville and stored at −80 °C until analysis.

### 2.3 Sample processing

Before analysis, the wastewater samples were thawed on ice, homogenized, and filtered through 0.22 μm polypropylene cellulose acetate centrifugal filters (25 mL, Thermo Fisher Scientific, MA, USA). A 1 mL filtrate aliquot was transferred to a 1.5 mL Eppendorf tube, followed by the addition of 50 μL of SILs at known concentrations. The samples were then lyophilized overnight at −50 °C and 0.133 mbar. The resulting residue was reconstituted to 50 μL with 40% MeOH, vortexed, and centrifuged at 10 °C for 5 min at 14 000 rpm. Finally, the supernatant was transferred to a liquid chromatography (LC) vial, and 10 μL were injected for LC-MS/MS analysis.

### 2.4 LC-MS/MS analysis

The samples were analyzed using a Waters Acquity H-class UPLC System coupled with a Waters Xevo TQ-S micro triple quadrupole mass spectrometer equipped with a CM-A Acquity (Waters, Milford, MA, USA). A sample was first loaded on an Acquity UPLC HSS T3 VanGuard pre-column (2.1 × 5 mm, 1.8 μm, Waters, MA, USA) with a six-port valve in position 1 at a flow rate of 0.2 mL min^−1^. The valve was then switched to position 2 at 1.5 min. The molecules trapped in the pre-column were further eluted onto a SunFire® C18 column (4.6 × 50 mm, 2.5 μm, Waters, MA, USA). The pre-column and column temperatures were set to 50 °C. Mobile phases A and B were composed of water and acetonitrile with 0.1% formic acid. The LC separation method is detailed in Table S1 and illustrated in Fig. S1.

Mass spectrometric (MS) detection was performed under the electrospray ionization positive mode (ESI^+^). Nitrogen and argon were used as the desolvation and collision gases, respectively. The MS parameters were set as follows: capillary voltage: 3.70 kV; cone voltage: 8–78 V; desolvation temperature: 350 °C; and desolvation gas flow rate: 650 L h^−1^.^24^ Collision energies optimized for each molecule ranged from 10 to 50 V (Table S2). LC-MS/MS data acquisition and processing were performed using MassLynx software (version 4.2; Waters, Milford, MA, USA). Peak integration was conducted using TargetLynx, a built-in MassLynx software component.

### 2.5 MRM method

Optimized MRM transitions were determined by analyzing both labeled and unlabeled standards in the ESI^+^ mode. For each analyte, the most abundant unique transition yielding an unlabeled daughter ion was selected for quantification. The second most abundant unique parent–daughter ion transition was monitored for confirmatory purposes. When multiple transitions exhibited similar intensities, the daughter ions with the higher *m*/*z* values were selected. Retention times and unique transitions used for quantification are listed in Table 2; the unique transitions used for confirmatory purposes are provided in Table S3.

**Table 2:** MRM transitions and retention times (RTs) used for the quantification of the target analytes, along with their corresponding stable isotope-labeled internal standards (SILs) used in the MRM method

Compound	Transition (*m*/*z*)	RT (min)	SIL	Transition (*m*/*z*)	RT (min)
Albuterol	240.278 → 148.077	3.71	Albuterol-d4	244.372 → 152.109	3.71
Amoxicillin	366.277 → 113.989	3.71	Amoxicillin-13C6	372.264 → 113.991	3.71
Azithromycin	749.884 → 116.059	4.26	Azithromycin-13Cd3	753.968 → 116.057	4.27
Budesonide	431.370 → 147.085	7.46	Budesonide-d8	439.420 → 147.166	7.41
Cetirizine	389.293 → 201.093	5.51	Cetirizine-d8	397.461 → 201.082	5.50
Diphenhydramine	256.328 → 167.130	4.87	Diphenhydramine-d6	262.368 → 167.126	4.87
Fexofenadine	502.500 → 466.469	5.13	Fexofenadine-d10	512.624 → 476.532	5.12
Fluticasone propionate	501.347 → 313.274	8.27	Fluticasone propionate-d5	506.377 → 313.315	8.27
Prednisolone	361.344 → 307.251	5.93	Prednisolone-d6	367.384 → 312.444	5.93
Prednisone	359.334 → 147.105	5.98	Prednisone-d8	367.384 → 149.177	5.91

### 2.6 Quality assurance and validation

Calibration curves were constructed using the mixtures of standards ranging from 0.05 to 50 ng mL^−1^. Linearity was accepted for regression coefficients (*R*^2^) exceeding 0.990. The detection limit (DL) was defined based on signal-noise ratio (S/N), as the lowest concentration at S/N ≥ 3. The quantification limit (QL) denoted the lowest concentration at S/N ≥ 10, with a 20% deviation from the true concentration.^14,24,25^

For quantification, the SILs were spiked into unlabeled standards, and response factors for each molecule were calculated:^26^


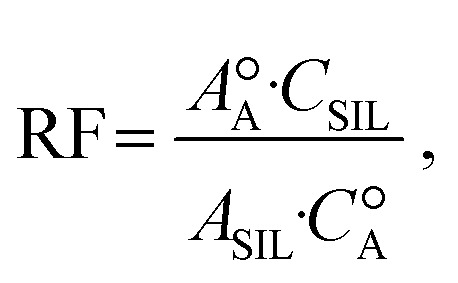
where 
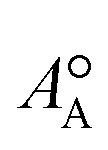
 and 
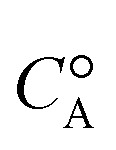
 are the peak area and concentration of the unlabeled standard A, respectively, and *A*_SIL_ and *C*_SIL_ are the peak area and concentration of the SIL.

Precision was assessed using intra- and inter-day repeatability assays. A pooled wastewater sample was prepared by combining 10 different wastewater samples spiked with the 10 SILs, lyophilized, and reconstituted in 50 μL of 40% MeOH, yielding a 20-fold concentration factor. The pooled sample was analyzed in triplicate on the same day and across three consecutive days. Precision was expressed as the relative standard deviation (RSD) of measured concentrations.

Matrix effects were assessed as follows by comparing the SILs peak areas in the pooled wastewater sample *versus* the peak areas of the SILs dissolved in ultrapure water:^24,27,28^
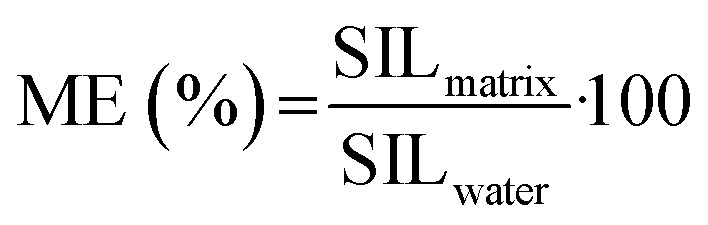


## 3. Results and discussion

**Fig. 1:**
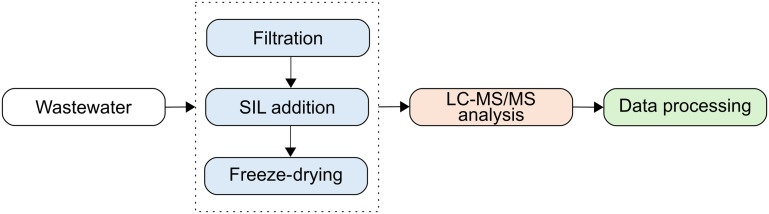
Representative workflow for the targeted LC-MS/MS method for quantifying respiratory pharmaceuticals in wastewater, including sample processing, LC-MS/MS analysis, and data processing.

Fig. 1 depicts the workflow of the developed method.

### 3.1 LC-MS/MS MRM method

**Fig. 2:**
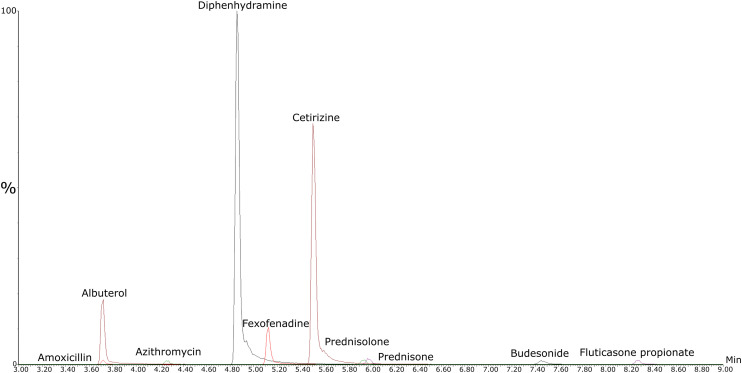
Representative selected ion chromatograms of the 10 target pharmaceuticals. Each trace corresponds to the quantifier ion transition optimized for sensitivity and selectivity, acquired through LC-MS/MS MRM.

Fig. 2 depicts a chromatogram of the 10 unlabeled standards. Most analytes achieved baseline separation in LC; however, two sets of analytes, namely, albuterol with amoxicillin and prednisolone with prednisone, co-eluted from the LC and entered MS together. Each pair of these molecules has different parent ion *m*/*z* values and daughter ions. Thus, they could still be differentiated and quantified in the first MRM quadrupole due to different parent ion selection.

The MRM transitions were selected to maximize the resolution and reliability for the identification and quantification of the target pharmaceuticals. Table 2 summarizes the MRM transitions and retention times (RTs) of the pharmaceuticals quantified in this work, along with the corresponding SILs. The MRM transitions included the precursor-to-product ion transitions (*m*/*z*) for both unlabeled and labeled compounds to ensure accurate quantification across the analyzed samples.

### 3.2 Method validation

The developed analytical method was validated according to the established guidelines to ensure its reliability for quantifying pharmaceuticals in wastewater.^26,29^Table 3 summarizes the main validation results. Individual peak area values are presented in Tables S4–S7.

**Table 3:** Method validation parameters for target compound quantification: linear range (ng mL^−1^), correlation coefficient (*R*^2^), detection (DL) and quantification limits (QL), and precision expressed as relative standard deviation (RSD, %), in a pooled *in situ* wastewater sample (*n* = 3) and across three days (*n* = 3). Matrix effects (*n* = 3) and recovery (%) were also evaluated

Compound	Linear range (ng mL^−1^)	*R* ^2^	DL (ng L^−1^)	QL (ng L^−1^)	Intraday (%, *n* = 3)	Inter-day (%, *n* = 3)	Matrix effect (%, *n* = 3)	Recovery (%)
Albuterol	0.10–49.99	0.998	1.2	5	1.59	5.9	64	114
Amoxicillin	0.38–37.60	0.999	19	94	4.48	<QL	66	82
Azithromycin	2.28–45.56	0.997	1.1	114	1.25	7.2	70	110
Budesonide	0.49–24.60	0.993	12.5	49	<DL	<DL	152	194
Cetirizine	0.21–41.90	0.999	1	10.5	1.55	1.9	84	87
Diphenhydramine	0.06–28.52	0.999	0.7	3	0.14	1.3	68	85
Fexofenadine	0.23–46.52	0.999	1.2	11.5	1.59	1.8	80	88
Fluticasone propionate	1.00–50.00	0.996	12.5	125	<DL	<DL	228	188
Prednisolone	0.49–12.34	0.999	12.5	50	<DL	<DL	88	82
Prednisone	1.00–24.90	0.996	12.5	50	1.97	3.6	98	113

Reported QL values for antihistamines such as cetirizine, diphenhydramine, and fexofenadine in previous studies on wastewater influent were comparable to those achieved using our method (0.2–4.7 ng L^−1^, 0.5 ng L^−1^, and 0.5–9.7 ng L^−1^, respectively).^22,27,30^ These molecules are usually present in high concentrations in wastewater, showing higher QL levels when analyzed by LC-MS/MS.

Matrix effects for antihistamines in the direct injection method have been reported to be 60–80% in wastewater.^27^ The matrix effects observed in this study varied substantially across the analytes, ranging from 64% to 228%. These effects arose from co-eluting impurities in wastewater that suppressed or enhanced the MS signal.^11,20,31,32^ Direct infusion generally induces stronger matrix effects during complex sample analyses. As reported, the developed method did not significantly reduce matrix effects; however, these effects were effectively compensated using SILs.

Prednisone exhibited the lowest matrix effect at 98%, whereas fluticasone propionate showed the highest (228%), followed by budesonide (152%). Despite this finding, fluticasone propionate was not detected in the *in situ* wastewater samples. Budesonide also displayed values lower than the QL. Overall, these results align with prior reports showing pronounced matrix effects in LC-MS/MS analysis of complex environmental samples.^27^ Importantly, SIL inclusion effectively compensated for these effects, as SILs undergo a similar signal suppression or enhancement to their corresponding analytes.^20,33^

### 3.3 Precision and recovery

The developed method demonstrated strong reproducibility. Intraday precision, assessed by analyzing a pooled wastewater sample in triplicate, ranged from 0.14% to 4.48%, while inter-day precision, based on the analyses across three consecutive days, ranged from 1.3% to 7.2%. Azithromycin exhibited greater variability due to its low abundance, while amoxicillin showed high intra- and inter-day variabilities with concentrations below the QL. This instability is consistent with the findings in prior reports on amoxicillin degradation during storage and analysis, leading to compromised reproducibility.^18^

The method provided 82–114% recoveries for the pharmaceuticals that were detected in wastewater, confirming that lyophilization is a robust and efficient preconcentration strategy for this analyte class in complex matrices.

### 3.4 Wastewater analysis

**Fig. 3:**
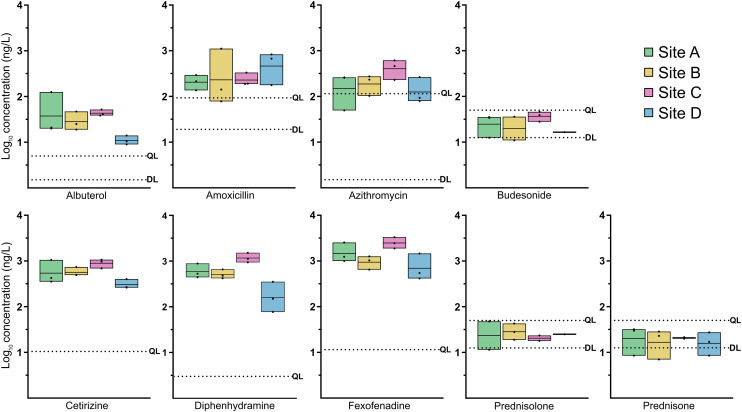
Detection limit (DL), quantification limit (QL), and concentration range of the nine pharmaceuticals quantified in *in situ* samples (*n* = 12; three samples from each of four sites in Louisville, KY) in log_10_ concentration (ng L^−1^).

The validated method was applied to 12 wastewater samples collected from four neighborhood catchments in Louisville, KY. Nine of the ten target pharmaceuticals were detected, and six (namely, albuterol, azithromycin, amoxicillin, cetirizine, diphenhydramine, and fexofenadine) were quantified with high confidence, *i.e.*, at concentrations above their QLs (Table S8, Fig. S2). These results demonstrate the utility of the method for surveilling a broad range of respiratory-related pharmaceuticals at the community scale. Fig. 3 displays the log_10_ concentrations (ng L^−1^) of the quantified pharmaceuticals across sites A–D, along with their respective DL and QL values.

### 3.5 Antihistamine occurrence

Antihistamines such as cetirizine, diphenhydramine, and fexofenadine are frequently detected in wastewater influent, with concentrations increasing over the past decade.^22,27,30,34,35^ In this study, fexofenadine was detected at the highest levels (417 to 3309 ng L^−1^), with the median concentration exceeding 1000 ng L^−1^. The cetirizine concentration ranged from 263 to 1070 ng L^−1^, and diphenhydramine from 78 to 1527 ng L^−1^, both with median values around 500 ng L^−1^. These findings are consistent with those of recent studies that demonstrated the persistence of these compounds in sewage.^22^ Fexofenadine and cetirizine have shown minimal degradation in sewage for 24 h.^36^

### 3.6 Antibiotic occurrence

Amoxicillin and azithromycin were consistently detected, with median concentrations of 203 and 245 ng L^−1^, respectively. Previous studies have reported higher amoxicillin levels in wastewater effluents (676–2560 ng L^−1^; mean > 1000 ng L^−1^).^11^ Azithromycin use sharply increased during 2019–2020, with consumption rising by ∼36% (ref. 37) and reported mean wastewater influent concentrations of 3700 ng L^−1^ during the COVID-19 pandemic.^38^ Azithromycin has broad applications for treating different infection types. It has also shown a high prevalence in other WBE studies. Additionally, the population's lack of knowledge might be a contributing factor when considering the incorrect use of antibiotics for treating cold and flu, consequently increasing the risk of antibiotic resistance. The detection of both antibiotics in our *in situ* samples and their routine presence in wastewater support their utility as indicators of community-level prescribing practices.^39,40^

### 3.7 Other pharmaceuticals

Albuterol was detected at relatively low concentrations of 9–126 ng L^−1^. Budesonide, prednisone, and prednisolone were detected only at levels below their respective QLs. Prior studies have reported higher budesonide (930–1340 ng L^−1^) and prednisone (18–353 ng L^−1^) concentrations in wastewater effluent.^11^ These discrepancies may reflect multiple factors, including lower prescription rates in the sampled catchments, excretion pattern variations, or more rapid degradation of these compounds in the wastewater matrix.

Similarly, fluticasone propionate, which was previously reported at 770–1550 ng L^−1^ (mean > 1000 ng L^−1^),^11^ was not detected herein. This nondetection might be caused by the low molecular abundance in the wastewater samples associated with poor ionization efficiency during MS analysis.^35^

### 3.8 Methodological considerations

The developed method demonstrates several strengths, including the ability to process volumes as small as 1 mL, high analytical precision, and effective matrix effect compensation through the SILs. Nevertheless, certain limitations warrant consideration. The interpretation of community-level pharmaceutical loads could be refined by normalizing the concentration to the population size, wastewater flow rate, and dilution factors. The degradation of pharmaceuticals during sewer transit or interactions with inhibitors may also influence analyte stability, suggesting the need for further investigation into compound-specific decay kinetics.

## 4. Conclusions

This study presents a robust and time-efficient LC-MS/MS with the MRM-validated method for quantifying 10 pharmaceuticals, associated with respiratory medication use, in wastewater. The developed method is the first to incorporate freeze-drying for preconcentration, online SPE for sample cleanup and reduced sample losses, and MRM for robust quantification. The use of stable isotope-labeled standards ensured accuracy and reproducibility in a complex matrix. The method demonstrated strong analytical performance, requiring only 1 mL of sample and achieving low detection limits. While this study focused on 10 respiratory-related pharmaceuticals, the developed method is readily extendable to other pharmaceuticals and metabolites. Its scalability and suitability for high-throughput analysis highlight its potential for broader application in WBE, enabling population-level surveillance of pharmaceutical usage and supporting public health efforts.

## Author contributions

S. B., T. S., A. B., R. H. H. and X. Z. conceived and designed research; R. L. G., and X. Y. performed experiments; X. Y., and R. L. G. analyzed data; R. L. G., X. Y., R. H. H. and X. Z. interpreted results of experiments; R. G. prepared the figures; R. L. G., R. H. H. and X. Z. drafted the manuscript; R. L. G., S. B., X. Y., T. S., A. B., R. H. H. and X. Z. edited and revised manuscript; R. L. G., S. B., X. Y., T. S., A. B., R. H. H. and X. Z. approved final version of manuscript.

## Conflicts of interest

There are no conflicts to declare.

## Supplementary Material

EW-012-D5EW00894H-s001

## Data Availability

The data generated in this study can be found in the published article and its supplementary information (SI) files. Supplementary information is available. See DOI: https://doi.org/10.1039/d5ew00894h.
