# Supplementary material for: Targeted LC-MS/MS method for quantifying respiratory pharmaceuticals in wastewater
Source: Environ Sci (Camb). 2025 Nov 28;12(2):554–62. doi: 10.1039/d5ew00894h (PMC12681241; doi:10.1039/d5ew00894h)

Supplementary Information (SI) for Environmental Science: Water Research & Technology.
This journal is © The Royal Society of Chemistry 2025

Supplemental Material

Title: Targeted LC-MS/MS Method for Quantifying Respiratory Pharmaceuticals in Wastewater

Running Title: Wastewater Analysis of Respiratory Pharmaceuticals

Regina L. Gasparetto[1,2], Scott Bickel[3,4], Xinmin Yin[1,2], Ted Smith[4], Aruni Bhatnagar[4], Rochelle H. Holm[4*], Xiang Zhang[1,2*]

[1] Department of Chemistry, University of Louisville, Louisville, Kentucky 40208, USA

[2] Center for Regulatory and Environmental Analytical Metabolomics, University of Louisville, Louisville, Kentucky 40292, USA

[3] Department of Pediatrics, University of Louisville, Louisville, Kentucky 40202, USA

[4] Christina Lee Brown Environment Institute, School of Medicine, University of Louisville, Louisville, Kentucky 40202, USA

Correspondence:

Dr. Rochelle H. Holm, Christina Lee Brown Environment Institute, School of Medicine, University of Louisville, Louisville, KY 40202, USA. Tel.: +1 502 852 5873. E-mail: rochelle.holm@louisville.edu.

Dr. Xiang Zhang, Department of Chemistry, University of Louisville, 2210 South Brook Street, Louisville, Kentucky 40208, USA. Tel.: +1 502 852 8878. E-mail: xiang.zhang@louisville.edu.

Table of Contents

Table S1. Gradient elution used for the chromatographic LC-MS/MS method. The table shows pumps 1 (quaternary solvent manager) and 2 (binary solvent manager). The gradient is represented by time (min), flow rate (mL/min), and percentage of mobile phases A (0.1% formic acid in water) and B (0.1% formic acid in acetonitrile) over the 20-min run. 3

Table S2. Multiple reaction monitoring (MRM) parameters used for the target analyte quantification. The table lists unlabeled and labeled analytes, selected precursor-to-product ion transitions (m/z), retention time (RT, min), dwell time (s), cone voltage (V), and collision energy (V) as optimized by the instrument. 6

Table S3. Unique parent–daughter ion transitions used for confirming target pharmaceuticals in wastewater samples. 7

Table S4. Evaluation of the matrix effects for each analyte. Matrix effects were calculated by comparing the response areas of the SIL spiked post-concentration into the matrix extracts versus neat solvent standards. The results are expressed as a percentage (%) signal suppression or enhancement. Values below 100% indicate ion suppression, while those above 100% signify ion enhancement. 8

Table S5. Intraday precision of the method for each analyte. The precision was assessed by analyzing the wastewater pooled sample (n = 3) under similar conditions on the same day. The results are expressed as RSD (%) of the measured concentrations. 9

Table S6. Inter-day precision of the method for each analyte. The precision was evaluated by analyzing the same sample across three days (*n* = 3). The results are expressed as RSD (%) of the measured concentrations, reflecting the reproducibility of the method over time. 10

Table S7. Method recoveries for each analyte. Recovery is the ratio of the measured concentration (ng/mL), obtained after processing the standard through the complete procedure, to the nominal concentration (ng/mL). The results are expressed in percentages. 11

Table S8. Pharmaceuticals quantified in in situ wastewater samples (*n* = 12) using the developed MRM method. Only nine pharmaceuticals could be quantified in the wastewater samples; fluticasone propionate was not detected. Reported area represents a quantitative parent–daughter ion transition. The presence of each molecule was confirmed using a confirmatory parent–daughter transition. 12

Figure S2. Pharmaceuticals quantified in in situ wastewater samples (n = 12) using the developed MRM method. Only nine pharmaceuticals could be quantified in the wastewater samples; fluticasone propionate was not detected. Concentrations are reported on a logarithmic scale at ng/L levels. 14

....

Table S1. Gradient elution used for the chromatographic LC-MS/MS method. The table shows pumps 1 (quaternary solvent manager) and 2 (binary solvent manager). The gradient is represented by time (min), flow rate (mL/min), and percentage of mobile phases A (0.1% formic acid in water) and B (0.1% formic acid in acetonitrile) over the 20-min run.

Pump 1 (QSM)

Time (min)	Flow rate (mL/min)	A (%)	B (%)
Initial	0.2	100	0
5.00	0.2	100	0
5.01	0.2	5	95
10.00	0.2	5	95
10.01	0.2	0	100
12.00	0.2	0	100
12.10	0.0	100	0
17.00	0.0	100	0
17.10	0.2	100	0
20.00	0.2	100	0

Pump 2 (BSM)

Time (min)	Flow rate (mL/min)	A (%)	B (%)
Initial	0.35	90	10
1.50	0.35	90	10
7.50	0.35	5	95
10.50	0.35	5	95
11.50	0.35	0	100
14.50	0.35	0	100
14.60	0.35	90	10
20.00	0.35	90	10

Pump 1 was used to load the sample onto an Acquity UPLC HSS T3 VanGuard pre-column (2.1 × 5 mm, 1.8 µm, Waters, MA, USA) with a 6-port valve in position 1 (Figure S1). The pharmaceuticals were trapped in the reversed phase pre-column while salts and other impurities, more attracted in the aqueous phase, were washed out with a high percentage of mobile phase A at a flow rate of 0.2 mL/min until 1.5 min. After the online cleanup step, the valve was changed to position 2 (Figure S1) and the molecules trapped in the pre-column were eluted onto a SunFire® C18 column (4.6 × 50 mm, 2.5 µm, Waters, MA, USA) using pump 2 with a linear gradient that

started at 10% B at 1.5 min and increased to 95% B at 7.5 min at a flow rate of 0.35 mL/min. The detailed gradient used in both pumps is described in Table S1. The pre-column and column temperatures were set to 50 °C.

Figure S1. Schematic representation of the Waters Xevo triple quadrupole (TQ) mass spectrometer in positions 1 and 2 of the two-dimensional chromatography system. The diagram illustrates the valve configuration and the flow paths during each switching cycle position.

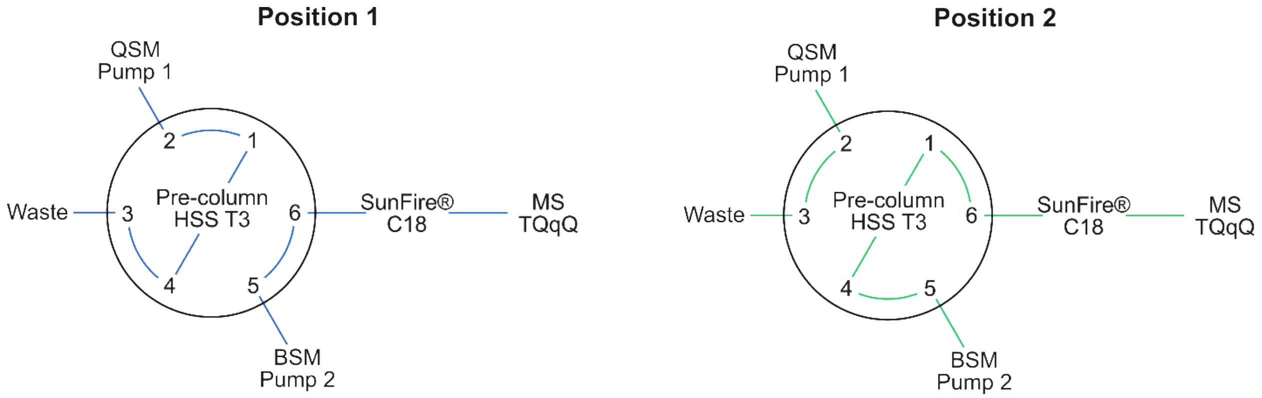

Table S2. Multiple reaction monitoring (MRM) parameters used for the target analyte quantification. The table lists unlabeled and labeled analytes, selected precursor-to-product ion transitions (m/z), retention time (RT, min), dwell time (s), cone voltage (V), and collision energy (V) as optimized by the instrument.

Analyte	Transition	RT (min)	Dwell (s)	Cone (V)	Collision (V)
Albuterol	240.278 → 148.077	3.71	0.025	32	18
Albuterol-d4	244.372 → 152.109	3.71	0.025	24	18
Amoxicillin	366.277 → 113.989	3.71	0.025	16	20
Amoxicillin-13C6	372.264 → 113.991	3.71	0.025	18	18
Azithromycin	749.884 → 116.059	4.26	0.025	78	50
Azithromycin-13Cd3	753.968 → 116.057	4.27	0.025	64	50
Budesonide	431.370 → 147.085	7.46	0.025	40	30
Budesonide-d8	439.420 → 147.166	7.41	0.025	20	30
Cetirizine	389.293 → 201.093	5.51	0.025	8	18
Cetirizine-d8	397.461 → 201.082	5.5	0.025	52	18
Diphenhydramine	256.328 → 167.130	4.87	0.025	22	12
Diphenhydramine-d6	262.368 → 167.126	4.87	0.025	18	10
Fexofenadine	502.500 → 466.469	5.13	0.025	76	26
Fexofenadine-d10	512.624 → 476.532	5.12	0.025	70	28
Fluticasone propionate	501.347 → 313.274	8.27	0.025	46	12
Fluticasone propionate-d5	506.377 → 313.315	8.27	0.025	30	14
Prednisolone	361.344 → 307.251	5.93	0.025	12	10
Prednisolone-d6	367.384 → 312.444	5.93	0.025	20	10
Prednisone	359.334 → 147.105	5.98	0.025	32	26
Prednisone-d8	367.384 → 149.177	5.91	0.025	28	26

Table S3. Unique parent–daughter ion transitions used for confirming target pharmaceuticals in wastewater samples.

Analyte	Confirmatory transition
Albuterol	240.278 → 166.096
Amoxicillin	366.277 → 208.115
Azithromycin	749.884 → 158.139
Budesonide	431.370 → 323.271
Cetirizine	389.293 → 165.907
Diphenhydramine	256.328 → 152.040
Fexofenadine	502.500 → 171.156
Fluticasone propionate	501.347 → 293.243
Prednisolone	361.344 → 325.267
Prednisone	359.334 → 267.252

Table S4. Evaluation of the matrix effects for each analyte. Matrix effects were calculated by comparing the response areas of the SIL spiked post-concentration into the matrix extracts versus neat solvent standards. The results are expressed as a percentage (%) signal suppression or enhancement. Values below 100% indicate ion suppression, while those above 100% signify ion enhancement.

Compound	Concentration (ng/mL)	MATRIX1	MATRIX2	MATRIX3	SIL1	SIL2	SIL3	**ME (%)**
Albuterol-d4	5.0	10403	9907	10332	15009	16565	16102	64
Amoxicillin-13C6	4.8	350	385	308	645	481	456	66
Azithromycin-13Cd3	5.0	716	476	528	759	826	867	70
Budesonide-d8	4.8	1313	1344	1315	953	808	849	152
Cetirizine-d8	4.2	70041	65217	62446	77348	81055	77660	84
Diphenhydramine-d6	4.4	119253	110494	104669	165039	161491	162133	68
Fexofenadine-d10	4.4	4297	4430	4240	5298	5065	5897	80
Fluticasone propionate-d5	4.6	1600	1431	1510	789	541	663	228
Prednisolone-d6	4.9	776	757	924	833	997	958	88
Prednisone-d8	49.0	393	393	331	370	379	394	98

Table S5. Intraday precision of the method for each analyte. The precision was assessed by analyzing the wastewater pooled sample (n = 3) under similar conditions on the same day. The results are expressed as RSD (%) of the measured concentrations.

	Rep 1			Rep 2			Rep 3			
Analyte	SIL area	Analyte	[ ] (ng/mL)	SIL area	Area	[ ] (ng/mL)	SIL area	Area	[ ] (ng/mL)	**RSD (%)**
Albuterol	9478	522	0.36	9941	550	0.36	8246	472	0.37	1.59
Amoxicillin	78	249	6.26	94	321	6.69	93	292	6.15	4.48
Azithromycin	744	196	2.12	431	111	2.08	611	162	2.13	1.25
Budesonide	< DL	< DL	< DL	< DL	< DL	< DL	< DL	< DL	< DL	-
Cetirizine	32727	33511	8.53	34464	36053	8.72	38311	40396	8.79	1.55
Diphenhydramine	97624	124059	7.36	93701	119256	7.37	96906	123014	7.35	0.14
Fexofenadine	2898	20434	29.33	3274	23066	29.3	3121	22605	30.13	1.59
Fluticasone propionate	< DL	< DL	< DL	< DL	< DL	< DL	< DL	< DL	< DL	-
Prednisolone	< DL	< DL	< DL	< DL	< DL	< DL	< DL	< DL	< DL	-
Prednisone	371	552	1.58	378	554	1.56	398	570	1.52	1.97

Table S6. Inter-day precision of the method for each analyte. The precision was evaluated by analyzing the same sample across three days (n = 3). The results are expressed as RSD (%) of the measured concentrations, reflecting the reproducibility of the method over time.

	Day 1			Day 2			Day 3			
Analyte	SIL area	Analyte	[ ] (ng/mL)	SIL area	Area	[ ] (ng/mL)	SIL area	Area	[ ] (ng/mL)	**RSD (%)**
Albuterol	9371	504	0.35	5069	301	0.39	5204	293	0.37	5.9
Amoxicillin	84	210	4.94	< QL	< QL	< QL	< QL	< QL	< QL	-
Azithromycin	498	142	2.31	538	141	2.16	342	137	2.49	7.2
Budesonide	< DL	< DL	< DL	< DL	< DL	< DL	< DL	< DL	< DL	-
Cetirizine	43048	43539	8.46	52038	54520	8.76	40229	41102	8.53	1.9
Diphenhydramine	92853	116508	7.27	88183	111477	7.32	65463	80711	7.14	1.3
Fexofenadine	3169	20080	26.37	3117	20406	27.34	2353	15250	26.97	1.8
Fluticasone propionate	< DL	< DL	< DL	< DL	< DL	< DL	< DL	< DL	< DL	-
Prednisolone	< DL	< DL	< DL	< DL	< DL	< DL	< DL	< DL	< DL	-
Prednisone	216	424	2.08	271	500	1.96	179	307	1.95	3.6

Table S7. Method recoveries for each analyte. Recovery is the ratio of the measured concentration (ng/mL), obtained after processing the standard through the complete procedure, to the nominal concentration (ng/mL). The results are expressed in percentages.

Compound	Std area	SIL area	Measured [ ] (ng/mL)	Nominal [ ] (ng/mL)	Recovery (%)
Albuterol	2350	13404	1.14	1.00	114
Amoxicillin	391	496	1.54	1.88	82
Azithromycin	190	308	5.01	4.56	110
Budesonide	451	1396	1.91	0.98	194
Cetirizine	37701	86029	3.64	4.19	87
Diphenhydramine	53607	135622	2.43	2.85	85
Fexofenadine	9347	4782	8.16	9.30	88
Fluticasone propionate	392	792	1.88	1.00	188
Prednisolone	261	1345	0.81	0.99	82
Prednisone	565	533	1.13	1.00	113

Table S8. Pharmaceuticals quantified in in situ wastewater samples (n = 12) using the developed MRM method. Only nine pharmaceuticals could be quantified in the wastewater samples; fluticasone propionate was not detected. Reported area represents a quantitative parent–daughter ion transition. The presence of each molecule was confirmed using a confirmatory parent–daughter transition.

	**Albuterol**				**Amoxicillin**				**Azithromycin**			
Site	Area	SIL Area	Measured [ ] (ng/mL)	Final [ ] (ng/L)	Area	SIL Area	Measured [ ] (ng/mL)	Final [ ] (ng/L)	Area	SIL Area	Measured [ ] (ng/mL)	Final [ ] (ng/L)
A	368	6127	0.39	19.50	360	165	4.27	213.50	594	922	5.19	259.50
A	585	9072	0.42	21.00	306	220	2.72	136.00	68	549	0.99	49.50
A	3038	7862	2.51	125.50	946	316	5.87	293.50	380	598	5.12	256.00
B	809	10522	0.50	25.00	107	135	1.56	78.00	284	417	5.50	275.00
B	555	9416	0.38	19.00	2615	231	22.23	1111.50	252	434	4.68	234.00
B	1238	8509	0.94	47.00	209	144	2.84	142.00	158	618	2.06	103.00
C	1369	10945	0.81	40.50	305	90	6.64	332.00	640	558	9.25	462.50
C	1155	9681	0.77	38.50	321	168	3.76	188.00	260	456	4.61	230.50
C	1688	10523	1.04	52.00	594	305	3.81	190.50	488	319	12.33	616.50
D	362	11414	0.21	10.50	2236	263	16.67	833.50	202	874	1.86	93.00
D	358	12850	0.18	9.00	1040	152	13.41	670.50	54	298	1.46	73.00
D	492	11238	0.28	14.00	347	192	3.54	177.00	161	246	5.26	263.00

	**Budesonide**				**Cetirizine**				**Diphenhydramine**			
Site	Area	SIL Area	Measured [ ] (ng/mL)	Final [ ] (ng/L)	Area	SIL Area	Measured [ ] (ng/mL)	Final [ ] (ng/L)	Area	SIL Area	Measured [ ] (ng/mL)	Final [ ] (ng/L)
A	210	1835	0.68	34.00	65352	63811	8.54	427.00	175067	94760	10.70	535.00
A	77	1786	0.25	12.50	63365	74577	7.08	354.00	168105	109009	8.93	446.50
A	215	1793	0.71	35.50	159306	62926	21.10	1055.00	284914	92960	17.75	887.50
B	-	-	-	ND	85130	71239	9.96	498.00	161217	109732	8.51	425.50
B	64	1754	0.22	11.00	85876	72753	9.84	492.00	178787	107623	9.62	481.00
B	151	1238	0.72	36.00	137021	77330	14.77	738.50	243361	106590	13.22	661.00
C	144	1527	0.56	28.00	123934	74740	13.82	691.00	357590	109408	18.92	946.00
C	174	1322	0.78	39.00	184223	71739	21.40	1070.00	540296	102471	30.53	1526.50
C	335	2152	0.92	46.00	176242	75661	19.41	970.50	450986	115858	22.54	1127.00
D	-	1694	-	ND	46424	72156	5.36	268.00	57563	110427	3.02	151.00
D	106	1903	0.33	16.50	40544	64384	5.25	262.50	31184	116812	1.55	77.50
D	-	1845	-	ND	79004	81890	8.04	402.00	145702	118974	7.09	354.50

	Fexofenadine				Prednisolone				Prednisone			
Site	Area	SIL Area	Measured [ ] (ng/mL)	Final [ ] (ng/L)	Area	SIL Area	Measured [ ] (ng/mL)	Final [ ] (ng/L)	Area	SIL Area	Measured [ ] (ng/mL)	Final [ ] (ng/L)
A	14261	2397	24.75	1237.50	-	-	-	ND	77	466	0.17	8.50
A	16362	3388	20.09	1004.50	58	1088	0.22	11.00	287	508	0.60	30.00
A	35989	2960	50.58	2529.00	203	879	0.96	48.00	207	345	0.64	32.00
B	16933	3419	20.60	1030.00	147	1089	0.56	28.00	56	437	0.14	7.00
B	11805	3796	12.93	646.50	92	1020	0.38	19.00	148	338	0.46	23.00
B	21026	3465	25.24	1262.00	196	948	0.86	43.00	289	536	0.57	28.50
C	32134	3542	37.73	1886.50	-	-	-	ND	149	400	0.40	20.00
C	40401	3438	48.88	2444.00	92	817	0.47	23.50	-	-	-	ND
C	53454	3360	66.18	3309.00	75	871	0.36	18.00	114	282	0.43	21.50
D	6940	3464	8.33	416.50	109	904	0.50	25.00	89	546	0.17	8.50
D	9824	3752	10.89	544.50	-	-	-	ND	237	454	0.55	27.50
D	19637	2791	29.27	1463.50	-	-	-	ND	148	460	0.34	17.00

ND = Non detected.

Figure S2. Pharmaceuticals quantified in in situ wastewater samples (n = 12) using the developed MRM method. Only nine pharmaceuticals could be quantified in the wastewater samples; fluticasone propionate was not detected. Concentrations are reported on a logarithmic scale at ng/L levels.

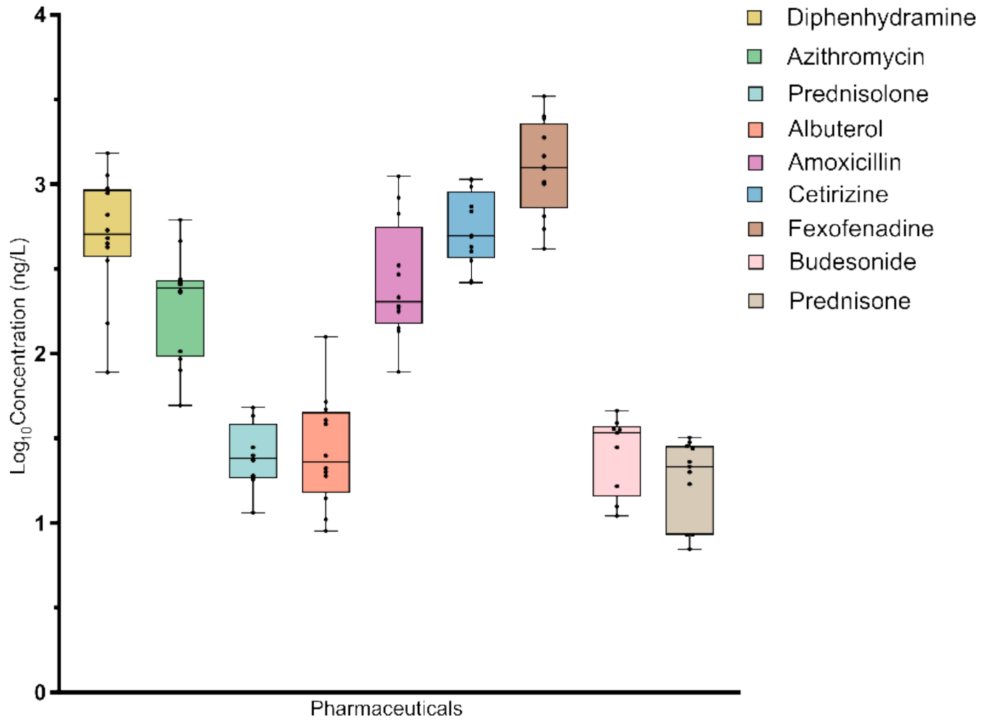

Supplement: EW-012-D5EW00894H-s001 [file EW-012-D5EW00894H-s001.pdf]
